# Metabolomics Analysis and Biosynthesis of Rosmarinic Acid in *Agastache rugosa* Kuntze Treated with Methyl Jasmonate

**DOI:** 10.1371/journal.pone.0064199

**Published:** 2013-05-28

**Authors:** Yeon Bok Kim, Jae Kwang Kim, Md. Romij Uddin, Hui Xu, Woo Tae Park, Pham Anh Tuan, Xiaohua Li, Eunsook Chung, Jai-Heon Lee, Sang Un Park

**Affiliations:** 1 Department of Crop Science, Chungnam National University, Daejeon, Republic of Korea; 2 National Academy of Agricultural Science, Rural Development Administration, Suwon, Republic of Korea; 3 College of Life Science, Inner Mongolia University for Nationalities, Inner Mongolia, China; 4 Department of Genetic Engineering, Dong-A University, Busan, Republic of Korea; Cankiri Karatekin University, Turkey

## Abstract

This study investigated the effect of methyl jasmonate (MeJA) on metabolic profiles and rosmarinic acid (RA) biosynthesis in cell cultures of *Agastache rugosa* Kuntze. Transcript levels of phenylpropanoid biosynthetic genes, i.e., *ArPAL*, *Ar4CL*, and *ArC4H*, maximally increased 4.5-fold, 3.4-fold, and 3.5-fold, respectively, compared with the untreated controls, and the culture contained relatively high amounts of RA after exposure of cells to 50 µM MeJA. RA levels were 2.1-, 4.7-, and 3.9-fold higher after exposure to 10, 50, and 100 µM MeJA, respectively, than those in untreated controls. In addition, the transcript levels of genes attained maximum levels at different time points after the initial exposure. The transcript levels of *ArC4H* and *Ar4CL* were transiently induced by MeJA, and reached a maximum of up to 8-fold at 3 hr and 6 hr, respectively. The relationships between primary metabolites and phenolic acids in cell cultures of *A. rugosa* treated with MeJA were analyzed by gas chromatography coupled with time-of-flight mass spectrometry. In total, 45 metabolites, including 41 primary metabolites and 4 phenolic acids, were identified from *A. rugosa*. Metabolite profiles were subjected to partial least square-discriminate analysis to evaluate the effects of MeJA. The results indicate that both phenolic acids and precursors for the phenylpropanoid biosynthetic pathway, such as aromatic amino acids and shikimate, were induced as a response to MeJA treatment. Therefore, MeJA appears to have an important impact on RA accumulation, and the increased RA accumulation in the treated cells might be due to activation of the phenylpropanoid genes *ArPAL*, *ArC4H*, and *Ar4CL*.

## Introduction

*Agastache rugosa* Kuntze, belonging to the mint family (Labiatae), is a perennial herb widely distributed in China, Japan, Korea, and Siberia. It is used in Chinese traditional medicine for the treatment of cholera, vomiting, and miasma, and has been reported to possess antitumor, antifungal, antiatherogenic, and cytotoxic activities [1]–[3]. Rosmarinic acid (RA) is an ester of caffeic acid and 3,4-dihydroxyphenyllactic acid found in plants. RA was initially isolated as a pure compound from the plant *Rosmarinus officinalis* [4] and is commonly found in the species Boraginaceae and the subfamily Nepetoideae of Lamiaceae [5]. RA exhibits a multitude of biological activities, including antiviral, antibacterial, anti-inflammatory, and antioxidant activities. Medicinal plants, herbs, and spices rich in RA are known for their beneficial effects on human health [6].

**Figure 1:**
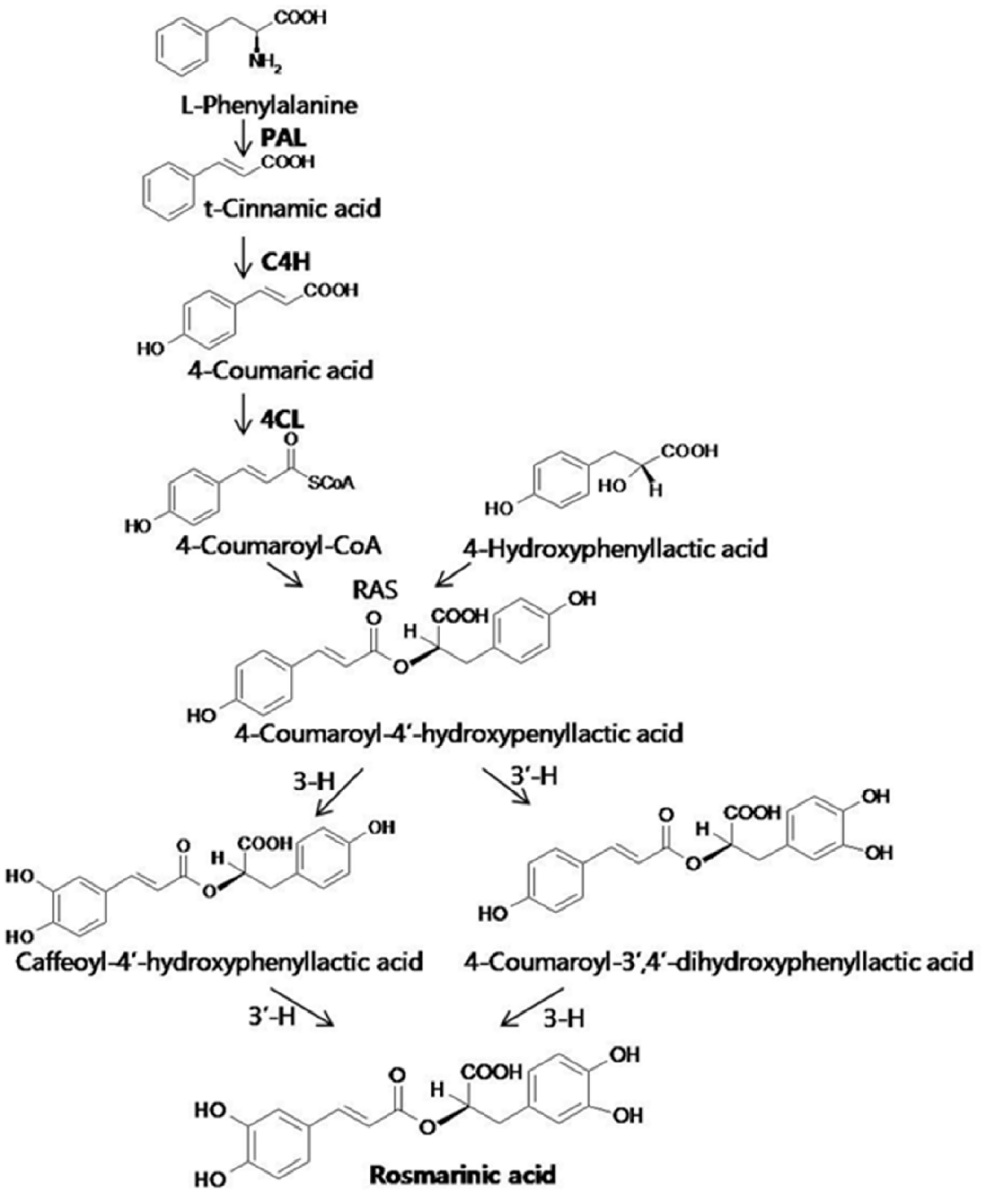
Proposed biosynthetic pathway of rosmarinic acid. PAL, phenylalanine ammonia-lyase; C4H, cinnamate 4-hydroxylase; 4CL, 4-coumaryl-CoA ligase; RAS, hydroxycinnamoyl-CoA:hydroxyphenyllactate hydroxycinnamoyl transferase.

RA is synthesized via the phenylpropanoid pathway (Figure 1). Phenylalanine ammonia lyase (PAL) catalyzes the conversion of phenylalanine to cinnamate. PAL also participates in the conversion of tyrosine to *p*-coumarate, albeit with lower efficiency [7]. Cinnamate 4-hydroxylase (C4H) catalyzes the synthesis of *p*-hydroxycinnamate from cinnamate, and 4-coumarate:coenzyme-A (CoA) ligase (4CL) converts *p*-coumarate to its coenzyme-A ester, activating it for reaction with malonyl CoA [8]. In the pathway leading to RA, condensation of 4-hydroxyphenyllactic acid with 4-coumaroyl-CoA is catalyzed by hydroxycinnamoyl-CoA:hydroxycinnamoyl transferase (RAS). In a final step, these reactions are catalyzed by two distinct cytochrome P450s: 3-hydroxycinnamoyl (3-H) and 3′ hydroxycinnamoyl (3′-H) [9].

RA is readily accumulated in undifferentiated plant cell cultures, and in some cases, its concentrations are much higher than those in the plant itself [10]. The first plant cell cultures found to accumulate RA were derived from *Coleus blumei* [10], [11].

In the plant biosynthetic pathway, phytohormone and microelement levels affect secondary metabolite accumulation in many *in vitro* plant cultures, but the most important factor is the elicitation process [12]. Methyl jasmonate (MeJA) and its associated free acid, jasmonic acid (JA), are important cellular regulators involved in diverse developmental processes, such as seed germination, root growth, fertility, fruit ripening, and senescence [13], [14]. Genes involved in JA biosynthesis, secondary metabolism, and cell-wall formation, and those encoding stress-protective and defense proteins are upregulated by MeJA treatment [15]. Recent studies have investigated the capability of JA and its methyl ester, MeJA, to induce the accumulation of various secondary metabolites [16]–[21].

Secondary metabolites are derived from central or primary metabolic processes in plants. The primary metabolite profile is closely related to the phenotype of the organism and includes important nutritional characteristics [22], [23]. In addition, metabolite profiling combined with chemometrics has proved to be useful in functional genomics research [24]. Metabolomics allows for classification of samples with diverse biological status, origin, or quality by using chemometric techniques such as hierarchical clustering analysis (HCA), and partial least squares discriminant analysis (PLS-DA). Therefore, in this study, we investigated the effects of MeJA on RA biosynthesis and expression of phenylpropanoid biosynthetic genes in the cell cultures of *A. rugosa*. Additionally, hydrophilic metabolic profiling (including phenolics) in *A. rugosa* cells by using gas chromatography time-of-flight mass spectrometry (GC-TOFMS) coupled with chemometrics was applied to determine the phenotypic variation and analyze content relationships.

## Materials and Methods

### Cell Suspension Culture Conditions

Cell cultures of *A. rugosa* were established from leaf explants-derived callus grown on MS medium containing 2 mg/L 2,4-dichlorophenoxyacetic acid (2,4-D) and 0.1 mg/L 6-Benzylaminopurine (BAP). Cell cultures were maintained in MS liquid medium and were sub-cultured at 14-day intervals. Cells were cultured at 120 rpm on a gyratory shaker at 25°C in a growth chamber under standard cool white fluorescent tubes with a flux rate of 35 µmol·s^−1^·m^−2^ and a 16 hr photoperiod. For biological replicates, three flasks were used for each individual and harvested them individually from each flask.

### Preparation and Application of MeJA

MeJA was dissolved in pure 100% ethanol to create stock solutions and then diluted as per experimental specification. To determine the concentrations of MeJA that promote maximum RA biosynthesis, different concentrations (0, 1, 10, 50, and 100 µM) of MeJA were added aseptically to the cultures at 10 days after cell inoculation for 4 days. The optimal concentration of 50 µM for induction of RA biosynthesis, was used to treat the cell culture harvested for metabolic profiling. A time course investigation of gene expression was conducted (0, 1, 3, 6, 12, 24, and 48 hr) using the optimal concentration of 50 µM MeJA. All cells were collected, frozen in liquid nitrogen, and stored at −80°C. The experiments were performed with three independent samples for biological replicates.

### Total RNA Isolation and Quantitative Real-time RT-PCR

Total RNA was isolated from cell cultures of *A. rugosa* using Plant total RNA mini kit (Geneaid, Taiwan) under the manufacturer’s instruction. The quality and concentration of total RNA was assessed by using a NanoVue Plus Spectrophotometer (GE Health Care Life Sciences, USA) and 1.2% formaldehyde RNA agarose gel. Subsequently, total RNA (1 µg) was reverse-transcribed with ReverTra Ace-α-(Toyobo, Osaka, Japan) Kit and oligo (dT)_20_ primer according to the manufacturer’s protocol. The gene expression levels of *ArPAL, ArC4H,* and *Ar4CL* were analyzed using a CFX96 real-time system (BioRad, Hercules, CA, USA). Gene-specific primers (Table 1) were used as previously described [25]. The SYBR Green quantitative real-time RT-PCR (qRT-PCR) assay was carried out in a total volume of 20 µl, containing 10 µl of 2X SYBR Green Real time PCR master mix (Toyobo, Osaka, Japan), 0.5 µM (each) of specific primers, and template cDNA was diluted 10-fold. PCR conditions were as follows: one cycle of 95°C for 3 min, followed by 40 cycles of 95°C for 15 s, 72°C for 20 s and annealing temperature (55°C). *Actin* was used as control (GenBank accession no. JX087435). For the comparison of *ArPAL, ArC4H,* and *Ar4CL* expression in samples treated with MeJA, the relative quantification of *ArPAL, ArC4H,* and *Ar4CL* expression was achieved by calibrating their transcript level against *Actin*. The reaction was performed in triplicate.

**Table 1:** List of real-time RT-PCR primers used in this study.

Primer name	Sequence (5′→3′)	Accession No.	Size (bp)
*ArActin*-RT (F)	ACCTCAAAATAGCATGGGGAAGT	JX087435	151
*ArActin*-RT (R)	GGCCGTTCTCTCACTTTATGCTA		
*ArPAL*-RT (F)	ACGGCTCCAACGGTCATAATAAT	AF326116	108
*ArPAL*-RT (R)	ATCCGCTTTACCTCCTCAAGGT		
*ArC4H*-RT (F)	GTTCGAGAGTGAGAATGATCCGT	AY616436	157
*ArC4H*-RT (R)	ATAATCCTTGAACAATTGCAGCC		
*Ar4CL*-RT (F)	ACATCTACTCGTTGAATTCGGTGC	AY587891	162
*Ar4CL*-RT (R)	AGTCGAAATTATCCACCAATGGA		

### HPLC Analysis of RA

RA content was analyzed according to the method described by Kim et al. [26] with a modification. Harvested cell cultures of *A. rugosa* were frozen in liquid N_2_, ground to a fine powder in mortar, and samples (1 g) were extracted with 80% methanol (10 ml) for 24 hr at 25°C. After centrifugation at 1000 × *g* for 5 min, the supernatant was filtered and reduced to dryness under vacuum dried, and dissolved in 1 ml methanol. The final extracts were filtered through a 0.45-µm PTFE syringe filter (Advantec DISMIC-13HP, Toyo Roshi Kaisha, Ltd., Tokyo, Japan) for high performance liquid chromatography (HPLC) analysis. The extracts were analyzed by HPLC on a C_18_ reverse phase column (4.6×250 mm; Ultrasphere, Beckman-Coulter) at room temperature. The solvent gradient used in this study was formed through with an initial proportion of mix of 70% solvent A (3% acetic acid in water) and 30% solvent B (methanol). After 50 minutes, the solvent gradient had reached 100% solvent B. The flow rate of the solvent was kept constant held at 1.0 ml min^−1^. Samples (20 µl) were detected at 280 nm. We identified the RA by matching the retention times and spectral characteristics to those from single HPLC run of a known RA standard.

### Metabolic Profiling

Polar metabolite extraction was performed as described previously [24]. A total of 10 mg of ground sample was extracted with 1 ml of a mixed solvent of methanol/water/chloroform (2.5∶1:1 by vol.). Ribitol solution (60 µl, 0.2 mg/ml) was added as an internal standard (IS). Extraction was performed at 37°C with a mixing frequency of 1200 rpm for 30 min, using a thermomixer compact (Eppendorf AG, Germany). The solutions were then centrifuged at 16,000 × *g* for 3 min. The polar phase (0.8 ml) was transferred into a new tube, and 0.4 ml of water was added before centrifugation in order to separate a nonpolar phase. The mixed contents of the tube were centrifuged at 16,000 **×** *g* for 3 min. The methanol/water phase containing hydrophilic metabolites was dried in a centrifugal concentrator (CVE-2000, Eyela, Japan) for 2 h, followed by a drying process in a freeze dryer for 16 h. Methoxime (MO)-derivatization was performed by adding 80 µl of methoxyamine hydrochloride (20 mg/ml) in pyridine and shaking at 30°C for 90 min. Trimethylsilyl (TMS) etherification was performed by adding 80 µl of MSTFA at 37°C for 30 min. GC-TOFMS was performed using an Agilent 7890A gas chromatograph (Agilent, Atlanta, GA, USA) coupled to a Pegasus HT TOF mass spectrometer (LECO, St. Joseph, MI). Derivatized sample (1 µL) was separated on a 30-m **×** 0.25-mm I.D. fused-silica capillary column coated with 0.25-µm CP-SIL 8 CB low bleed (Varian Inc., Palo Alto, CA, USA). The split ratio was set at 1∶25. The injector temperature was 230°C. The helium gas flow rate through the column was 1.0 mL/min. The temperature program was as follows: Initial temperature 80°C for 2 min, followed by an increase to 320°C at 15°C/min, and a 10 min hold at 320°C. The transfer line and ion-source temperatures were 250 and 200°C, respectively. The scanned mass range was 85–600 *m/z*, and the detector voltage was set at 1700 V.

### Statistical Analysis

For qRT-PCR statistical analysis, the data were analyzed by the computer software Statistical Analysis System (SAS version 9.2). All data are given as the mean and standard deviation of triplicate experiments. Treatment mean comparisons were performed with the Least Significant Difference (LSD). The relative quantification data acquired from GC-TOFMS was subjected to PLS-DA (SIMCA-P version 12.0; Umetrics, Umeå, Sweden) to evaluate the relationships in terms of similarity or dissimilarity among groups of multivariate data. The PLS-DA output consisted of score plots to visualize the contrast between different samples and loading plots to explain the cluster separation. The data file was scaled with unit variance scaling before all variables were subjected to the PLS-DA.

## Results and Discussion

### Inducible Expression of Phenylpropanoid Biosynthetic Genes in Cell Culture of *A. rugosa* with Different Concentrations of MeJA

**Figure 2:**
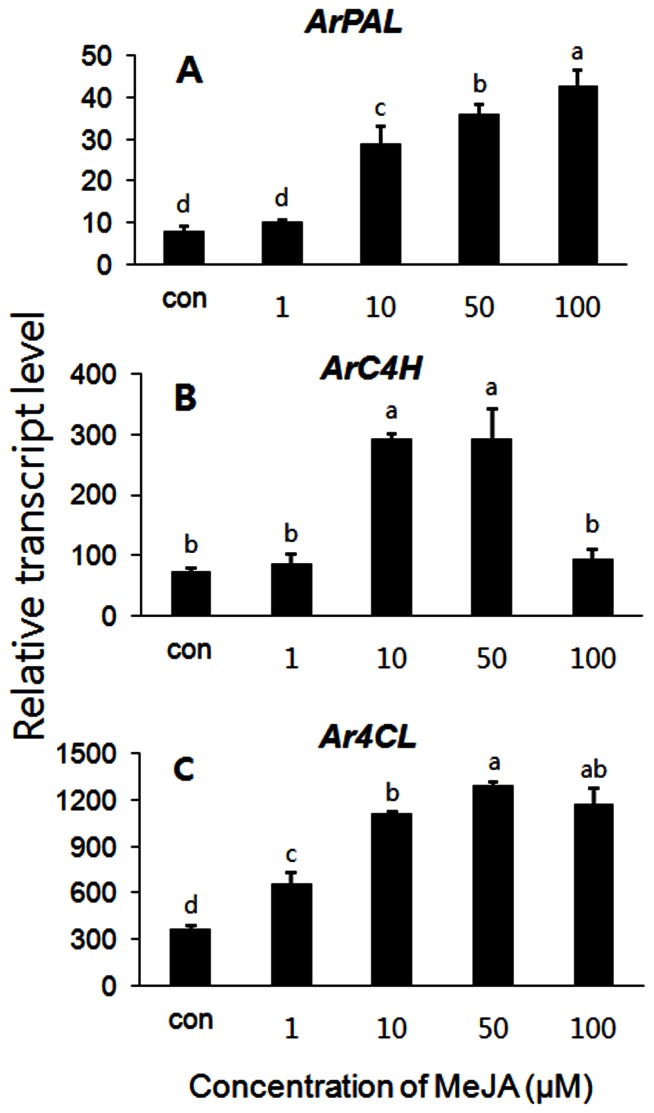
Transcript levels after treatment with methyl jasmonate in suspension cell culture of *A. rugosa.* Inducible transcript levels of (A) *ArPAL*, (B) *ArC4H*, and (C) *Ar4CL* when treated with different concentrations of MeJA. The longitudinal axis indicates the expression levels of genes relative to that of *actin*. Each value is the mean of three replicates, and error bars indicate SDs. Values represent LSD (p = 0.05).

To investigate the effects of MeJA on the inducible expression of phenylpropanoid biosynthetic genes in the cell culture of *A. rugosa*, cells were initially cultured for 5 days in MS basal media supplemented with 2 mg/L 2,4-D and 0.1 mg/L BAP and then treated with different concentrations of MeJA. Transcript levels of the *ArPAL*, *ArC4H* and *Ar4CL* phenylpropanoid biosynthetic genes increased with increasing MeJA concentration, and the transcript level of phenylpropanoid biosynthetic genes reached a maximum at 50 µM MeJA (Figure 2). Transcript levels vary with concentrations of MeJA in different species. Previously it was reported that the expression levels of *AgPAL* and *AgC4H* in the suspension cells of *Angelica gigas* were the highest at 300 µM MeJA and these levels are associated with the production of secondary metabolites [27]. At 50 µM of MeJA-treated cells, the transcript levels of the *ArPAL*, *Ar4CL*, and *ArC4H* phenylpropanoid biosynthetic genes were increased 4.5-, 3.4- and 3.5-fold, respectively, compared with the untreated control. The transcript levels of *Ar4CL* were much higher showing 36.0 and 4.4 times higher than those of *ArC4H* and *ArPAL*, respectively at 50 µM MeJA (Figure 2). In another study, Yamamura et al. [28] and Tsuruga et al. [29] described that the activity of PAL, the entry point enzyme for the phenylpropanoid pathway was rapidly up-regulated after MeJA treatment in the *Lithospermum erythrorhizon* cell suspension cultures. The peak of LePAL activity exhibited about 11-fold higher in the MeJA-treated cells than in the control cells [29]. Therefore, Krzyzanowska et al. [30] suggested that enhanced PAL level will correspond to enhanced RA production.

### Effect of Different Concentrations of MeJA on RA Accumulation

**Figure 3:**
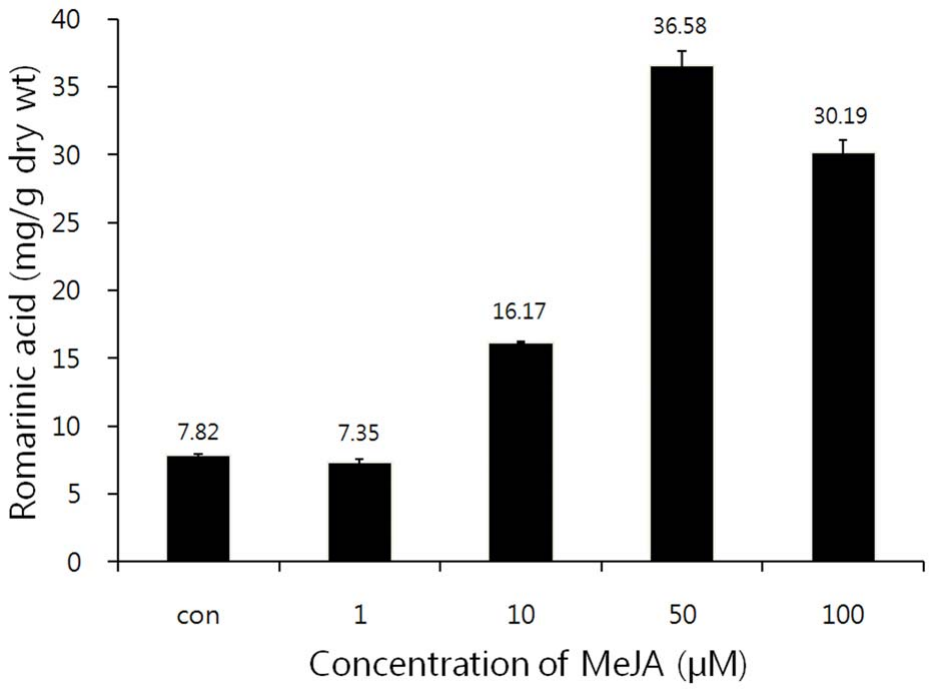
Time courses of rosmarinic acid production induced by application of MeJA with different concentrations in the suspension cell culture of *A. rugosa*. Each value is the mean of three replicates, and error bars indicate SDs.

The dose-response experiment indicated that RA content varied widely depending on the concentrations of exogenous MeJA (Figure 3). Levels of RA accumulation increased with increasing MeJA concentrations up to 50 µM, and then began to decline. RA levels were 2.1-, 4.7-, and 3.9-fold higher compared to untreated control, after exposure to 10, 50, and 100 µM of exogenous MeJA, respectively (Figure 3). Cell suspension cultures derived from various plant species have shown that elicitation enhances the production of secondary metabolites. In the yeast extract-treated *L. erythrorhizon* cells suspension cultures, RA content increased after a lag period of 8 hr and then showed a maximum at 24 hr after treatment [31], MeJA strongly induced 10-fold RA accumulation compared with control cells [32]. Hakkim et al. [33] reported that sucrose (5.0%), phenylalanine (0.25 g/L), yeast extract (0.5 g/L), and MeJA (100 M) resulted in 2.7-, 4.1-, 7.0-, and 8.6-fold increases in RA content, respectively, compared with control cells, in *Ocimum sanctum* cell cultures. Jeong and Park [34] described several abiotic elicitors capable of enhancing growth and ginseng saponin biosynthesis in the hairy roots of *Panax ginseng.* Moreover, MeJA was highly effective in inducing both *trans*- and *cis*-resveratrol accumulation in *Vitis vinifera* cell suspension cultures [35]. Ketchum et al. [36] reported that cell suspension cultures of *Taxus canadensis* and *Taxus cuspidata* rapidly produced paclitaxel (Taxol) and other taxolids in response to elicitation with MeJA. In this study, RA content was the highest in the cell suspensions treated with 50 µM MeJA (Figure 3) after 3 hr of culturing. In contrast, the addition of 100 µM MeJA within 24 hr was the most effective dose to result the highest RA content in the *Mentha piperita* suspension culture [30].

### The Effect of Exposure Length at Optimal MeJA on Expression of Phenylpropanoid Biosynthetic Genes in Cell Culture of *A. rugosa*

**Figure 4:**
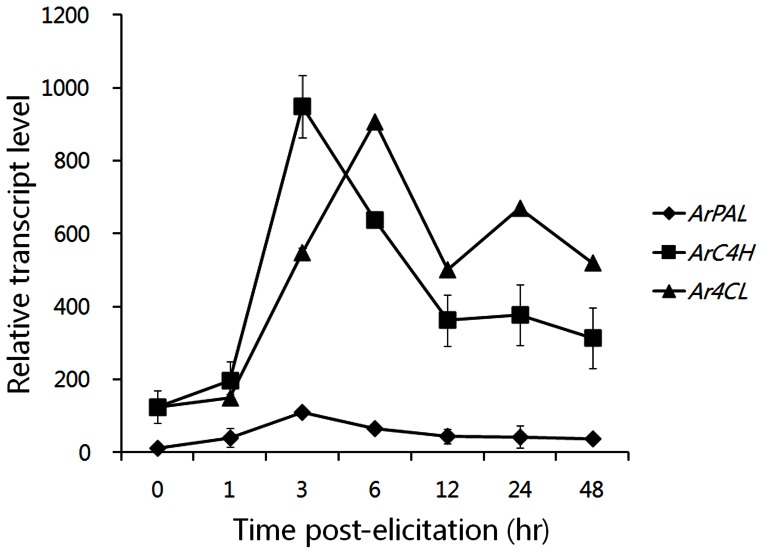
Time course of induction of phenylpropanoid biosynthetic genes by application of MeJA at 50 µM in the suspension cell culture of *A. rugosa*. The longitudinal axis indicates the expression levels of genes relative to that of *actin*. Each value is the mean of three replicates, and error bars indicate SDs.

Transcript levels of the phenylpropanoid pathway genes *ArPAL*, *Ar4CL*, and *ArC4H* leading to RA biosynthesis in *A. rugosa* suspension culture increased after exposure to MeJA at 50 µM. The extent to which the levels of transcript were affected by MeJA was related to the time of exposure of the *A. rogosa* suspension culture to jasmonates. As shown in Figure 2, the transcript levels of *ArPAL*, *Ar4CL*, and *ArC4H* reached maximum values at 50 µM MeJA. The accumulation of *ArPAL*, *Ar4CL*, and *ArC4H* transcriptions was monitored over 0, 1, 3, 6, 12, 24, and 48 hr periods treated with 50 µM MeJA. Transcripts of different RA biosynthetic genes responded differently to MeJA exposure. Shortly after the application of MeJA, transcript levels were comparable to those of the control, although higher levels were observed at later time points (Figure 4). The time following initial exposure when transcript levels were highest differed among the genes. In the case of the *ArPAL* and *ArC4H* genes, the highest level of transcript accumulation occurred 3 hr after initial exposure to 50 µM MeJA. At 3 hr, levels of *ArPAL* and *ArC4H* transcripts were 9.2- and 7.8-fold higher, respectively, than in the control at 0 hr (Figure 4). The largest increase in transcript accumulation was apparent for the *Ar4CL* gene 6 hr after MeJA treatment, when the *Ar4CL* transcript was 7.4-fold more abundant than that in control at 0 hr (Figure 4).

Our observation is supported by previous work of Mizukami et al. [32], who observed that phenylalanine ammonia-lyase (PAL) and 4-hydroxyphenylpyruvate reductase (HPPR) activities were induced rapidly and transiently during RA accumulation, whereas tyrosine aminotransferase (TAT) activity exhibited only a slight increase in *L. erythrorhizon* cell suspension cultures. Recently, our group reported that the high transcript levels of *ArPAL*, *ArC4H*, and *Ar4CL* may explain the high values of the flavonoid, acacetin, and its derivative, tilianin, in the flowers and leaves of *A. rugosa* [25]. The expressions of phenylpropanoid biosynthetic genes (*PAL*, *C4H*, and *4CL*) were reported to correlate with the flavonoid content in several plants [36]–[40]. Park et al. [27] pointed out that MeJA plays an important role in the elicitation procedure, causing either immediate or circuitous activation of the genes involved in secondary metabolism.

The results of this study indicate that the addition of MeJA induced increased transcript levels of *ArPAL*, *ArC4H*, and *Ar4CL*, and enhanced RA accumulation in cell suspension cultures, compared with the control.

### GC-TOFMS Analysis of Polar Metabolites

In this study, low-molecular-weight molecules from *A. rugosa* cells were identified by GC-TOFMS. ChromaTOF software was used to assist with peak location. Peak identification was performed by comparison with reference compounds and the use of an in-house library. In addition, identification of amino acids, sugars, and phenolic acids was performed using direct comparison of the sample mass chromatogram with mass chromatograms of commercially available standard compounds, which were obtained by a similar MO/TMS derivatization and GC-TOFMS analysis. In total, 45 metabolites, including 19 amino acids, 16 organic acids, 7 sugars, 2 sugar alcohols, and a single amine, were detected in *A. rugosa* cells (Figure S1). Of these, 4 phenolics (ferulic, *p*-coumaric, *p*-hydroxybenzoic, and salicylic acid) were identified in the samples. Quantification was performed using selected ions (Table S1), and the quantitative calculations of all analytes were based on the peak area ratios relative to that of the IS (Table S2).

**Figure 5:**
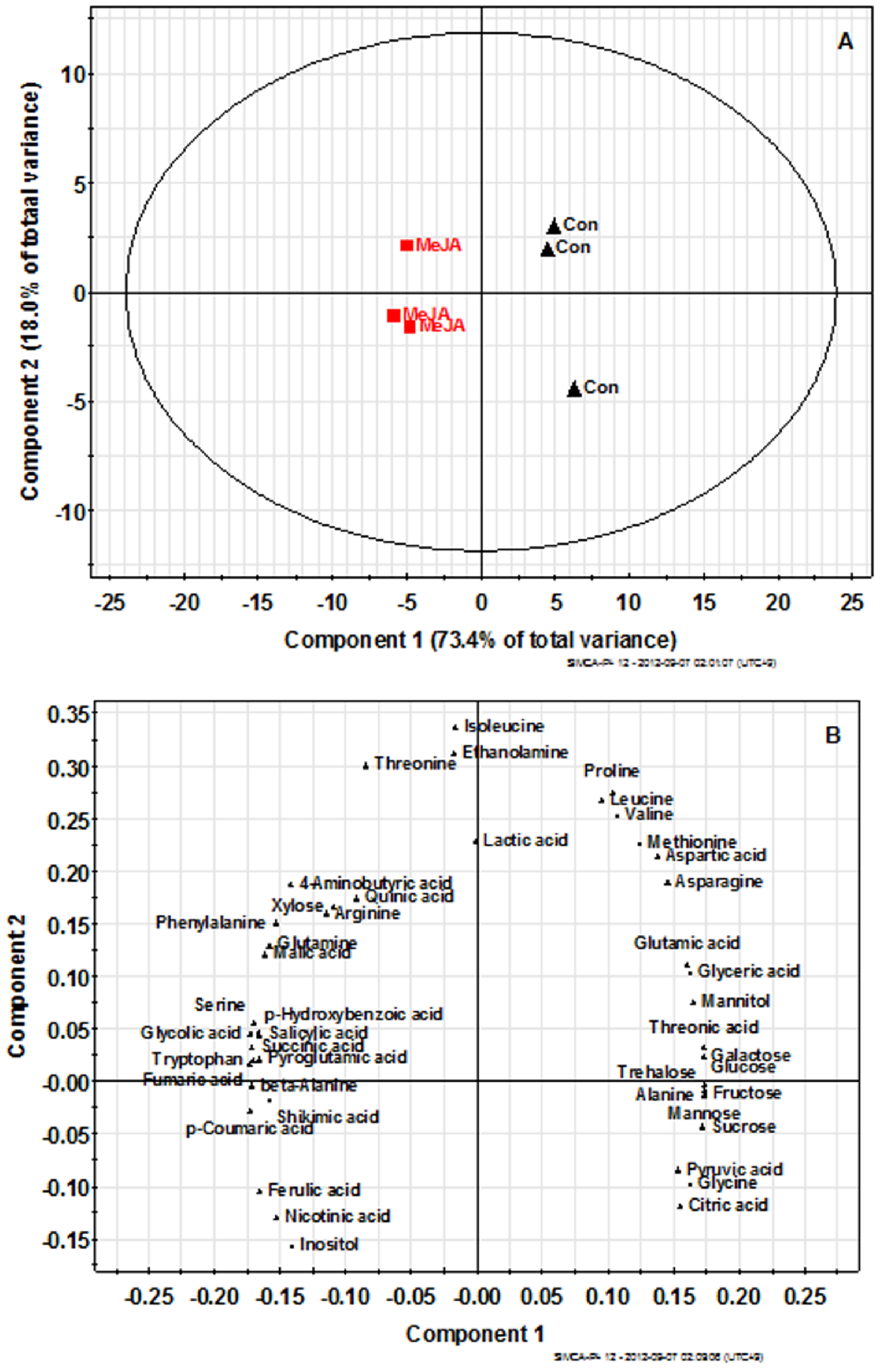
Scores (A) and loading plots (B) of principal components 1 and 2 of the PLS-DA results obtained from polar metabolite data of the MeJA-treated *A. rugosa* cells and control cells.

Forty-five metabolites were subjected to PLS-DA to identify differences between the metabolite profiles of MeJA-treated cells and controls (Figure 5). The loading plot of the PLS-DA reveals the magnitude and direction of correlation of the original variables with principal components. All phenolic acids as well as aromatic amino acids were clustered in the right of the loading plot, while threonic acid and sugars such as fructose, galactose, mannose, and sucrose were clustered in the left of the loading plot. The first principal component resolved the metabolite profiles of MeJA-treated cells and control cells and accounted for 73% of the variation, whereas the second component accounted for an additional 17% of the variation. The results indicated that compared to the control group, the level of aromatic amino acids and phenolic acids contents were increased, whereas threonic acid and sugars were decreased after treatment with MeJA. Similar results were reported by Liang et al. [41] where in *Brassica rapa* leaves treated with MeJA, hydroxycinnamates, and glucosinolate were elevated and glucose, sucrose, and amino acids decreased. In another study, Broeckling et al. [42] also reported lower sucrose levels in MeJA- and yeast-elicited samples than those in controls, suggesting that at least some of the effects observed in the primary metabolic pool are a consequence of fundamental metabolic repartitioning of carbon resources rather than specific induction elicited by a stimulus.

Our results revealed correlations between metabolites that participate in similar pathways and demonstrated the robustness of the present experimental system. Furthermore, in addition to secondary metabolites, several primary metabolites such as β-alanine, GABA and succinic acid accumulated after MeJA elicitation. The accumulation of these metabolites cannot be explained by their ecological functions or through common catabolic phenomena such as protein degradation. β-alanine, GABA, and succinic acid levels were elevated in MeJA-treated cells [42].

**Figure 6:**
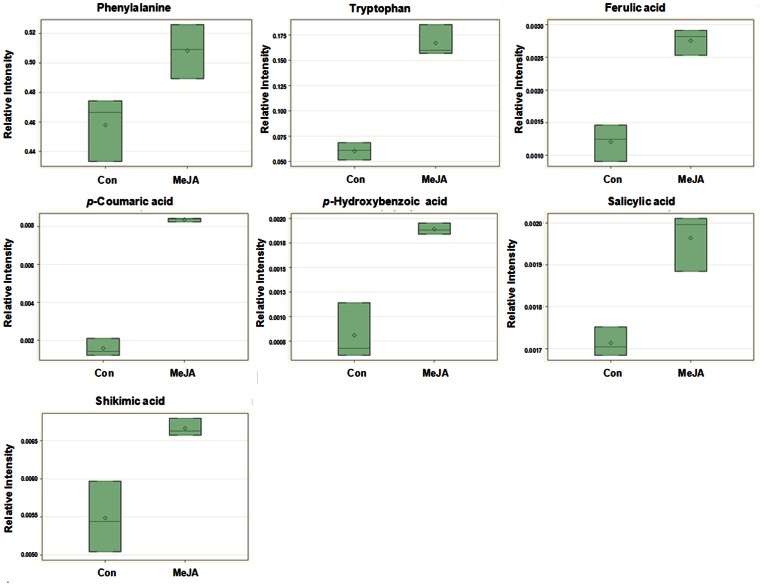
Box plots of aromatic amino acids, phenolic acids, and shikimic acid that were significantly different between the MeJA-treated *A. rugosa* cells and control cells.

The levels of all aromatic amino acids, phenolic acids, and shikimic acid were higher in MeJA-treated cells than in the control cells (Figure 6). It has been reported that the aromatic amino acids phenylalanine, tyrosine, and tryptophan in plants are synthesized via the shikimate pathway, and the branched aromatic amino acids biosynthesis pathway both serve as precursors of numerous of secondary metabolites such as pigments, alkaloids, hormones, and cell wall components [43]. The aromatic amino acids of [^11^C]Tyrosine, [^11^C]phenylalanine, and [^11^C]tryptophan were increased by 1.5-, 12-, and 12-fold, respectively, compared to controls, at 4 h after MeJA treatment in *Nicotiana tabacum* [44]. This observation suggested that MeJA could cause rapid changes in [^11^C]photosynthate pools, thus favoring increased partitioning of new carbon into amino acids, whereas MeJA could also impart selective control over the shikimate pathway, giving rise to differential partitioning of new carbon into its metabolites.

### Conclusion

Using GC-TOFMS, we were able to distinguish changes in the levels of a wide range of metabolites such as aromatic amino acids, phenolic acids, and shikimic acids in MeJA-treated cultures of *A. rugosa*. Phenolic acids and aromatic amino acids are produced from the shikimate pathway. The induction of both aromatic amino acids and phenolic acids was observed in cells as a response to MeJA treatment. This observation suggests that metabolomics can assist in dissecting mechanisms regulating conversion of primary to secondary metabolism in plants. The expression of transcripts involved in the phenylpropanoid biosynthetic pathway in addition to the changes in RA and various primary metabolites increased after MeJA treatment compared to controls, indicating that MeJA elicitor induces differential transcriptional and metabolic re-programming in suspension cultures of *A. rugosa*. Therefore, future studies should be aimed at further improving RA compound production in cell suspension cultures of *A. rugosa* used in the pharmaceuticals.

## Supporting Information

Figure S1 **Selected ion chromatograms of metabolites extracted from** ***A. rugosa*** **as MO/TMS derivatives separated on a 30 m×0.25 mm I.D. fused silica capillary column coated with 0.25 µm CP-SIL 8 CB low-bleed stationary phase.** Peak identification: 1 pyruvic acid, 2 lactic acid, 3 valine, 4 alanine, 5 glycolic acid, 3′ valine, 6 serine, 7 ethanolamine, 8 leucine, 9 isoleucine, 10 proline, 11 nicotinic acid, 12 glycine, 13 succinic acid, 14 glyceric acid, 15 fumaric acid, 6′ serine, 16 threonine, 17 *β*-alanine, 18 malic acid, 19 salicylic acid, 20 aspartic acid, 21 methionine, 22 pyroglutamic acid, 23 4-aminobutyric acid, 24 threonic acid, 25 arginine, 26 glutamic acid, 27 phenylalanine, 28 p-hydroxybenzoic acid, 29 xylose, 30 asparagine, 31 glutamine, 32 shikimic acid, 33 citric acid, 34 quinic acid, 35 fructose, 35′ fructose, 36 galactose, 37 glucose, 38 mannose, 39 mannitol, 40 *p*-coumaric acid, 41 inositol, 42 ferulic acid, 43 tryptophan, 44 sucrose, 45 trehalose, IS internal standard (ribitol).
(DOCX)

Table S1 **The detected chromatographic and spectrometric data of the 45 identified compounds analyzed by GC-TOFMS.**
(DOCX)

Table S2 **Forty-five metabolite contents in MeJA-treated** ***A. rugosa*** **cells.**
(DOCX)
