# Supplementary material for: Metabolomics Analysis and Biosynthesis of Rosmarinic Acid in Agastache rugosa Kuntze Treated with Methyl Jasmonate
Source: PLoS One. 2013 May 28;8(5):e64199. doi: 10.1371/journal.pone.0064199 (PMC3665811; doi:10.1371/journal.pone.0064199)
Supplement: Figure S1 — Selected ion chromatograms of metabolites extracted from A. rugosa as MO/TMS derivatives separated on a 30 m×0.25 mm I.D. fused silica capillary column coated with 0.25 µm CP-SIL 8 CB low-bleed stationary phase. Peak identification: 1 pyruvic acid, 2 lactic acid, 3 valine, 4 alanine, 5 glycolic acid, 3′ valine, 6 serine, 7 ethanolamine, 8 leucine, 9 isoleucine, 10 proline, 11 nicotinic acid, 12 glycine, 13 succinic acid, 14 glyceric acid, 15 fumaric acid, 6′ serine, 16 threonine, 17 β-alanine, 18 malic acid, 19 salicylic acid, 20 aspartic acid, 21 methionine, 22 pyroglutamic acid, 23 4-aminobutyric acid, 24 threonic acid, 25 arginine, 26 glutamic acid, 27 phenylalanine, 28 p-hydroxybenzoic acid, 29 xylose, 30 asparagine, 31 glutamine, 32 shikimic acid, 33 citric acid, 34 quinic acid, 35 fructose, 35′ fructose, 36 galactose, 37 glucose, 38 mannose, 39 mannitol, 40 p-coumaric acid, 41 inositol, 42 ferulic acid, 43 tryptophan, 44 sucrose, 45 trehalose, IS internal standard (ribitol). (DOCX) [file pone.0064199.s001.docx]

**
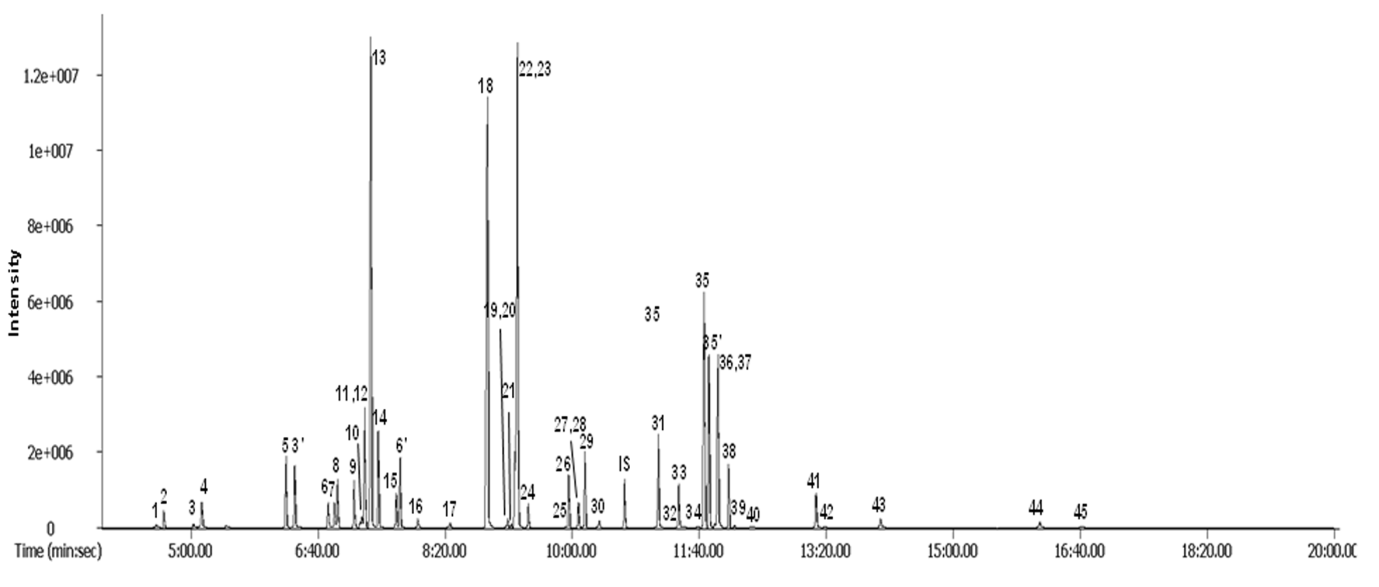
**

**Figure S1.** **Selected ion chromatograms of metabolites extracted from *A. rugosa* as MO/TMS derivatives separated on a 30 m × 0.25 mm I.D. fused silica capillary column coated with 0.25 μm CP-SIL 8 CB low-bleed stationary phase.** Peak identification: 1 pyruvic acid, 2 lactic acid, 3 valine, 4 alanine, 5 glycolic acid, 3’ valine, 6 serine, 7 ethanolamine, 8 leucine, 9 isoleucine, 10 proline, 11 nicotinic acid, 12 glycine, 13 succinic acid, 14 glyceric acid, 15 fumaric acid, 6’ serine, 16 threonine, 17 *β*-alanine, 18 malic acid, 19 salicylic acid, 20 aspartic acid, 21 methionine, 22 pyroglutamic acid, 23 4-aminobutyric acid, 24 threonic acid, 25 arginine, 26 glutamic acid, 27 phenylalanine, 28 p-hydroxybenzoic acid, 29 xylose, 30 asparagine, 31 glutamine, 32 shikimic acid, 33 citric acid, 34 quinic acid, 35 fructose, 35’ fructose, 36 galactose, 37 glucose, 38 mannose, 39 mannitol, 40 *p*-coumaric acid, 41 inositol, 42 ferulic acid, 43 tryptophan, 44 sucrose, 45 trehalose, IS internal standard (ribitol).
