# Supplementary material for: Metabolomics Analysis and Biosynthesis of Rosmarinic Acid in Agastache rugosa Kuntze Treated with Methyl Jasmonate
Source: PLoS One. 2013 May 28;8(5):e64199. doi: 10.1371/journal.pone.0064199 (PMC3665811; doi:10.1371/journal.pone.0064199)
Supplement: Table S1 — The detected chromatographic and spectrometric data of the 45 identified compounds analyzed by GC-TOFMS. (DOCX) [file pone.0064199.s002.docx]

**Table S1.** The detected chromatographic and spectrometric data of the 45 identified compounds analyzed by GC-TOFMS.

| Compound | RT^a^ | RRT^b^ | Mass fragment^c^ | Quantification ion^d^ |
| --- | --- | --- | --- | --- |
| Pyruvic acid | 4.32 | 0.415 | 115, 174, 189 | 174 |
| Lactic acid | 4.38 | 0.421 | 117, 147, 191 | 147 |
| Valine | 5.02 | 0.482 | 130, 146, 156 | 146 |
| Alanine | 5.08 | 0.488 | 116, 147, 190 | 116 |
| Glycolic acid | 6.15 | 0.591 | 147, 177, 205 | 147 |
| Valine | 6.22 | 0.598 | 144, 156, 218 | 144 |
| Serine | 6.48 | 0.622 | 116, 132, 147 | 116 |
| Ethanolamine | 6.52 | 0.626 | 100, 147, 174 | 174 |
| Leucine | 6.55 | 0.629 | 102, 147, 158 | 158 |
| Isoleucine | 7.08 | 0.680 | 147, 158, 218 | 158 |
| Proline | 7.13 | 0.685 | 142, 158, 216 | 142 |
| Nicotinic acid | 7.15 | 0.687 | 106, 136, 180 | 180 |
| Glycine | 7.17 | 0.689 | 147, 174, 248 | 174 |
| Succinic acid | 7.22 | 0.693 | 129, 147, 247 | 147 |
| Glyceric acid | 7.27 | 0.698 | 133, 147, 189 | 147 |
| Fumaric acid | 7.42 | 0.713 | 143, 147, 245 | 245 |
| Serine | 7.45 | 0.716 | 147, 204, 218 | 204 |
| Threonine | 7.59 | 0.729 | 101, 117, 219 | 219 |
| β-Alanine | 8.23 | 0.791 | 147, 174, 248 | 174 |
| Malic acid | 8.53 | 0.819 | 147, 233, 245 | 147 |
| Salicylic acid | 9.09 | 0.873 | 135, 149, 267 | 267 |
| Aspartic acid | 9.09 | 0.873 | 100, 147, 232 | 100 |
| Methionine | 9.12 | 0.876 | 128, 147, 176 | 176 |
| Pyroglutamic acid | 9.15 | 0.879 | 147, 156, 230 | 156 |
| 4-Aminobutyric acid | 9.17 | 0.881 | 147, 174, 304 | 174 |
| Threonic acid | 9.25 | 0.889 | 147, 205, 220 | 147 |
| Arginine | 9.55 | 0.917 | 142, 147, 162 | 142 |
| Glutamic acid | 9.57 | 0.919 | 128, 156, 246 | 246 |
| Phenylalanine | 10.05 | 0.965 | 100, 192, 218 | 218 |
| *p*-Hydroxybenzoic acid | 10.06 | 0.966 | 193, 223, 267 | 223 |
| Xylose | 10.10 | 0.970 | 103, 147, 217 | 103 |
| Asparagine | 10.21 | 0.981 | 116, 132, 231 | 116 |
| Ribitol | 10.41 | 1.000 | 103, 147, 217 | 217 |
| Glutamine | 11.08 | 1.064 | 147, 156, 245 | 156 |
| Shikimic acid | 11.17 | 1.073 | 147, 204, 255 | 204 |
| Citric acid | 11.24 | 1.080 | 147, 273, 347 | 273 |
| Quinic acid | 11.39 | 1.094 | 147, 255, 345 | 345 |
| Fructose | 11.44 | 1.099 | 103, 147, 217 | 103 |
| Fructose | 11.48 | 1.103 | 103, 147, 217 | 103 |
| Galactose | 11.52 | 1.107 | 147, 205, 319 | 147 |
| Glucose | 11.55 | 1.109 | 147, 160, 205 | 147 |
| Mannose | 12.03 | 1.156 | 147, 205, 319 | 147 |
| Mannitol | 12.08 | 1.160 | 147, 217, 319 | 319 |
| *p*-Coumaric acid | 12.21 | 1.173 | 219, 249, 293 | 219 |
| Inositol | 13.12 | 1.260 | 147, 217, 305 | 305 |
| Ferulic acid | 13.19 | 1.266 | 308, 323, 338 | 338 |
| Tryptophan | 14.03 | 1.348 | 202, 219, 348 | 202 |
| Sucrose | 16.08 | 1.545 | 147, 217, 361 | 217 |
| Trehalose | 16.41 | 1.576 | 147, 191, 361 | 191 |

^a^Retention time (min).

^b^Relative retention time (retention time of analyte/retention time of ribitol).

^c^Lists of first three ions with the highest intensity. Ions in boldface indicate the most intense product ion.

^d^Specific mass ion used for quantification.
