# Supplementary material for: Metabolomics Analysis and Biosynthesis of Rosmarinic Acid in Agastache rugosa Kuntze Treated with Methyl Jasmonate
Source: PLoS One. 2013 May 28;8(5):e64199. doi: 10.1371/journal.pone.0064199 (PMC3665811; doi:10.1371/journal.pone.0064199)
Supplement: Table S2 — Forty-five metabolite contents in MeJA-treated A. rugosa cells. (DOCX) [file pone.0064199.s003.docx]

**Table S2. Forty-five metabolite contents in MeJA-treated *A. rugosa* cells.**

| Compound | Control cells | MeJA-treated cells |
| --- | --- | --- |
| Pyruvic acid | 1.00 ± 0.06 | 0.88 ± 0.01 |
| Lactic acid | 1.00 ± 0.10 | 0.98 ± 0.08 |
| Valine | 1.00 ± 0.12 | 0.83 ± 0.12 |
| Alanine | 1.00 ± 0.12 | 0.07 ± 0.01 |
| Glycolic acid | 1.00 ± 0.06 | 1.35 ± 0.02 |
| Serine | 1.00 ± 0.07 | 1.41 ± 0.08 |
| Ethanolamine | 1.00 ± 0.09 | 1.01 ± 0.09 |
| Leucine | 1.00 ± 0.23 | 0.76 ± 0.17 |
| Isoleucine | 1.00 ± 0.22 | 1.01 ± 0.15 |
| Proline | 1.00 ± 0.34 | 0.64 ± 0.09 |
| Nicotinic acid | 1.00 ± 0.04 | 1.20 ± 0.09 |
| Glycine | 1.00 ± 0.12 | 0.72 ± 0.02 |
| Succinic acid | 1.00 ± 0.02 | 1.10 ± 0.01 |
| Glyceric acid | 1.00 ± 0.06 | 0.74 ± 0.02 |
| Fumaric acid | 1.00 ± 0.06 | 1.60 ± 0.04 |
| Threonine | 1.00 ± 0.11 | 1.07 ± 0.07 |
| β-Alanine | 1.00 ± 0.03 | 1.37 ± 0.04 |
| Malic acid | 1.00 ± 0.05 | 1.12 ± 0.01 |
| Salicylic acid | 1.00 ± 0.02 | 1.15 ± 0.04 |
| Aspartic acid | 1.00 ± 0.06 | 0.87 ± 0.04 |
| Methionine | 1.00 ± 0.09 | 0.84 ± 0.08 |
| Pyroglutamic acid | 1.00 ± 0.09 | 1.37 ± 0.11 |
| 4-Aminobutyric acid | 1.00 ± 0.05 | 1.07 ± 0.02 |
| Threonic acid | 1.00 ± 0.01 | 0.73 ± 0.01 |
| Arginine | 1.00 ± 0.18 | 1.19 ± 0.11 |
| Glutamic acid | 1.00 ± 0.02 | 0.88 ± 0.04 |
| Phenylalanine | 1.00 ± 0.05 | 1.11 ± 0.04 |
| *p*-Hydroxybenzoic acid | 1.00 ± 0.36 | 2.34 ± 0.07 |
| Xylose | 1.00 ± 0.12 | 1.12 ± 0.07 |
| Asparagine | 1.00 ± 0.11 | 0.75 ± 0.04 |
| Glutamine | 1.00 ± 0.28 | 1.67 ± 0.13 |
| Shikimic acid | 1.00 ± 0.08 | 1.21 ± 0.02 |
| Citric acid | 1.00 ± 0.04 | 0.92 ± 0.02 |
| Quinic acid | 1.00 ± 0.11 | 1.08 ± 0.09 |
| Fructose | 1.00 ± 0.07 | 0.35 ± 0.01 |
| Galactose | 1.00 ± 0.02 | 0.34 ± 0.02 |
| Glucose | 1.00 ± 0.05 | 0.38 ± 0.02 |
| Mannose | 1.00 ± 0.08 | 0.21 ± 0.01 |
| Mannitol | 1.00 ± 0.01 | 0.88 ± 0.02 |
| *p*-Coumaric acid | 1.00 ± 0.30 | 5.33 ± 0.06 |
| Inositol | 1.00 ± 0.03 | 1.08 ± 0.02 |
| Ferulic acid | 1.00 ± 0.23 | 2.28 ± 0.16 |
| Tryptophan | 1.00 ± 0.13 | 2.77 ± 0.26 |
| Sucrose | 1.00 ± 0.16 | 0.20 ± 0.01 |
| Trehalose | 1.00 ± 0.05 | 0.51 ± 0.03 |

Values are expressed relative to control cells and presented as mean ± SD of determinations from three independent samples.
